# Treatment of gingival recession type 1 using coronally advanced flap with leucocytes-platelet rich fibrin: a randomized controlled trial

**DOI:** 10.1007/s00784-026-06899-4

**Published:** 2026-05-07

**Authors:** Nuno Bernardo Malta dos Santos, Gustavo Vicentis Oliveira Fernandes, Tiago Marques, Manuel Correia Sousa, André Correia, María Pilar Batalla Vázquez, Juan Blanco-Carrión

**Affiliations:** 1 Faculty of Dental Medicine, Universidade Católica Portuguesa, Viseu, 3504-505 Portugal; 2 University of Santiago de Compostela, Santiago de Compostela, Spain; 3 Center for Interdisciplinary Research in Health, Universidade Católica Portuguesa, Viseu, 3504-505 Portugal; 4 A. T. Still University - Missouri School of Dentistry & Oral Health, 1500 Park Av., St. Louis, MO 63104 USA; 5 Periodontology Unit, School of Medicine and Dentistry, University of Santiago de Compostela, Praza do Obradoiro, s/n, Santiago de Compostela, 15782 Spain; 6 Odontología Médico-Quirúrgica (OMEQUI) Research Group, Health Research Institute of Santiago de Compostela (IDIS), Santiago de Compostela, Spain

**Keywords:** Gingival recession, Platelet-Rich Fibrin, Digital assessment, Root coverage, Periodontal plastic surgery

## Abstract

**Objective:**

This study aimed to evaluate the clinical outcomes of gingival recession Type 1 (RT1) treatment using a coronally advanced flap (CAF) alone and in combination with L-PRF at 6 months.

**Materials and methods:**

A total of 70 RT1 from 19 patients were included. Participants were randomly assigned to the test group (TG, CAF + L-PRF, *n* = 42) and the control group (CG) (CAF alone, *n* = 28). Clinical parameters were assessed at baseline and at 6 months: the primary outcomes were percentage of root coverage (%RC) and complete root coverage (CRC); and the secondary outcomes included mean root coverage (MRC), changes in gingival thickness (GT) and volumetric tissue gain, recession area reduction, analgesic consumption, healing quality (the Inflammatory Proliferative Remodeling [IPR] score), and patient-reported outcome measures (PROMs). Statistical analyses were performed to determine differences between groups; a random-intercept mixed-effects model was used for all site-level outcomes.

**Results:**

At six months, %RC was 89.30% ± 20.33% (TG) and 81.60% ± 27.93% (CG) (*p* > 0.05). For the TG and CG, respectively, %CRC was 73.81% and 57.14%; the mean GT gain was 0.16 ± 0.10 mm and 0.11 ± 0.10 mm (*p* = 0.08); the mean volume gain was 1.13 ± 1.25 mm^3^ and 0.86 ± 0.84 mm^3^ (*p* = 0.32); the mean number of pills taken was 1.67 ± 0.98 and 2.25 ± 1.02 (*p* = 0.04); the esthetic satisfaction was 95% ± 5% and 90% ± 7% (*p* = 0.31); willingness to undergo the procedure again: 93% ± 4% and 88% ± 6% (*p* = 0.22); sensitivity reduction: 85% ± 6% and 80% ± 8% (*p* = 0.19). There was no statistically significant difference in healing quality and PROMs.

**Conclusion:**

Both CAF alone and CAF combined with L-PRF yielded comparable clinical and volumetric outcomes in RT1 treatments. No statistically significant advantages were observed with the adjunctive use of L-PRF.

**Clinical relevance:**

Because CAF alone achieves high predictability for RT1 defects, the routine adjunctive use of L-PRF provides limited additional clinical benefits.

**Supplementary Information:**

The online version contains supplementary material available at 10.1007/s00784-026-06899-4.

## Introduction

The demand for cosmetic and minimally invasive dental treatments has increased markedly in recent years. In periodontology, gingival recession (GR) represents a frequent clinical challenge due to its esthetic impact and associated functional consequences, including dentinal hypersensitivity, susceptibility to root caries, and difficulties in plaque control [1, 2]. GR is highly prevalent and age-dependent, often progressing over time [3]. Its etiology is multifactorial [2, 4], involving both anatomical factors, such as a thin gingival phenotype, and behavioral factors, such as traumatic toothbrushing, necessitating surgical intervention to restore the gingival margin position and improve clinical outcomes [5, 6].

Among available techniques, the coronally advanced flap (CAF) and its modified version (mCAF) are widely regarded as the treatment of choice for multiple recession types I and II (RT1 and RT2). Although subepithelial connective tissue grafts (sCTG) [7, 8] remain the gold standard due to their high predictability, they are associated with donor-site morbidity. They may compromise patient satisfaction due to excessive gingival thickening [9, 10]. Consequently, Leukocyte-Platelet Rich Fibrin (L-PRF) has gained clinical interest as a biologically driven adjunct [11–13]. As a second-generation autologous platelet concentrate, L-PRF provides a fibrin scaffold enriched with platelets and growth factors that promote angiogenesis, fibroblast proliferation, and soft-tissue healing [14, 15]. 

The combination of CAF and L-PRF has been proposed to enhance root coverage (RC) by increasing gingival thickness (GT) and accelerating healing [13, 16]. However, evidence from randomized controlled trials (RCTs) remains inconsistent, with results ranging from outcomes comparable to those with CAF alone to only marginally superior [17, 18]. This uncertainty is often attributed to variability in preparation protocols and the limitations of traditional assessment methods. Variability in L-PRF preparation protocols, rapid degradation, and limited long-term data further contribute to this uncertainty [19]. Crucially, the subtle biological effects of L-PRF may be overlooked by conventional periodontal probing, which is limited by millimetric resolution and examiner variability.

Recent advances in digital technologies, including three-dimensional and volumetric analysis, offer a novel opportunity for precise and objective evaluation of soft-tissue outcomes. By explicitly framing digital analysis within the study rationale, this trial aims to use higher-resolution methods to detect submillimetric gains that conventional linear measurements may miss. Such well-designed RCTs remain essential for clarifying the clinical benefits of L-PRF and supporting evidence-based decision-making [13].

Therefore, the objective of this randomized controlled trial (RCT) was to compare the efficacy of CAF alone versus CAF combined with L-PRF in treating RT1 GRs. The primary outcomes were the percentage of root coverage (%RC) and complete root coverage (CRC). Secondary outcomes included mean root coverage (MRC), percentage of root coverage by area (%RC-area), changes in keratinized tissue width (KTW), GT, volumetric (VoL) gain, postoperative healing, and patient-reported outcome measures (PROMs). The null hypothesis was that the adjunctive use of L-PRF would not yield statistically superior outcomes compared with CAF alone.

## Materials and methods

This RCT, with two arms and a parallel design, adhered to the Declaration of Helsinki (1964, updated 2024) [20] and the regulations and Standards for Good Clinical Practice. It was designed in accordance with the CONSORT guidelines (http://www.consort-statement.org/) and approved by the local Ethics Committee at Universidade Católica Portuguesa (Viseu, Portugal; n. 2020 − 123). Additionally, it was registered at ClinicalTrials.gov (NCT06591156). Patients attending the *Clínica Dentária Universitária* of the Universidade Católica Portuguesa (Viseu, Portugal) received an explanation and an evaluation. They signed the informed consent form, which described various procedures, including those with and without blood collection. The recruitment and follow-up took place from September/2020 to December/2023.

### Sample size calculation

Kuka et al.’s [21] data reported a MRC of 88.36% (Standard Deviation [SD] = 15.45%) for CAF + L-PRF and 74.63% (SD = 8.05%) for CAF alone; these data were used to calculate the sample size. An effect size of approximately 1.12 was determined. Aiming for a statistical power of 0.80 and a significance level (α) of 0.05, the required sample size per group was determined. Given that each patient may have multiple recession sites, the sample size calculation was adjusted to account for clustering using an intraclass correlation coefficient (ICC) of 0.1 and an average of 2 recession sites per patient. Then, 10 patients per group were required, yielding a total sample size of 20. This corresponded to approximately 20 recession sites per group, for a total of 40 in the study. It is worth noting that some patients may have more than 2 GRs. This adjustment enables the detection of clinically significant differences while accounting for potential dropouts and variability.

### Eligibility criteria

The sample was chosen based on the following inclusion criteria: (1) patients over 18 years old; (2) non-smokers or smokers of less than ten cigarettes; (3) no medical pathology that prevents the application of treatments affects the response of these and the healing process (protein and vitamin deficiency, therapeutic radiation, metabolic disorders - diabetes, hypercalcemia, and others), (4) no drug (antimetabolic, immunosuppressive) or hormonal disorder, (5) at least the presence of two neighboring teeth, (6) GRs Miller class 1/RT1 in the anterior upper maxilla (central and lateral incisors, canine) or first and second premolars, (7) and presence of oral health verified with O’Leary plaque control record (PCR) [22].

The exclusion criteria were: (1) pregnancy, (2) presence of carious lesions, occlusal trauma, or previous RC procedures, (3) presence of a prosthetic crown on experimental teeth, (4) bleeding on probing (BoP) ≥ 25% [23], (5) gingival recessions present with ≤ 1.5 mm of keratinized tissue (KT) apical to the recession area, (6) probing pocket depth (PD) > 3 mm, and interproximal CAL > 0 mm, (7) allergy to some component/material applied in this study, (8) reject blood collection, and (9) does not be present after 6 months for re-evaluation.

Participants were withdrawn if they: (1) requested to leave the study; (2) failed to attend scheduled follow-ups; (3) developed systemic conditions affecting healing; or (4) failed to maintain adequate plaque control.

### Groups and randomization process

Two groups composed this study: (1) Control group: only CAF; and (2) Test group: CAF + L-PRF. Participants were randomly assigned to groups using a computer-generated randomization sequence. The randomization method, sequence generation, was performed on Randomization.com using randomly permuted blocks [24]. This plan was created using sed 2,948, with the same number of subjects per block/number of blocks as initially entered, and the same treatment labels.

One study collaborator (FA) performed random generation. Concealment was conducted by the same clinician who was not involved in participant care or data collection. This collaborator (FA) concealed the table used to assign participants to the appropriate group. Before the intervention, the blinded investigator (NBMS) contacted the collaborator, who instructed the patient to be assigned to either the test or control group. It was not possible to blind the participants because blood was collected for L-PRF preparation; they should be aware of their group allocation and accept it. The outcome evaluator was completely blinded to group assignments to ensure the assessment of clinical outcomes remained unbiased.

### Primary and secondary outcomes

Clinical parameters were assessed at baseline and six months. The primary outcomes were the %RC based on recession depth (Re) and %CRC. Secondary outcomes included MRC, KTW gain, and reduction in recession area; superimposing pre- and postoperative digital models quantified GT and VoL gains. Specific metrics included maximum and mean GT gains at defined regions of interest (ROI-1 and ROI-2) using a 3D comparison protocol [17, 25]. Healing quality was evaluated using the Inflammatory, Proliferative, and Remodeling (IPR) scale to grade tissue maturation objectively.

### Patient-reported outcome measures (PROMs)

PROMs were collected using validated tools to assess morbidity and satisfaction [26, 27]. Postoperative pain was monitored and quantified using a 10-cm Visual Analog Scale (VAS) at 5 and 14 days, and analgesic consumption (600 mg ibuprofen protocol) was recorded at 5 and 14 days, with total pill consumption recorded. Additional PROMs focused on subjective satisfaction at 6 months enabled patients to complete questionnaires on esthetic satisfaction, willingness to repeat the procedure, and changes in cervical dentin hypersensitivity (CDH).

### Digital workflow

**Fig. 1:**
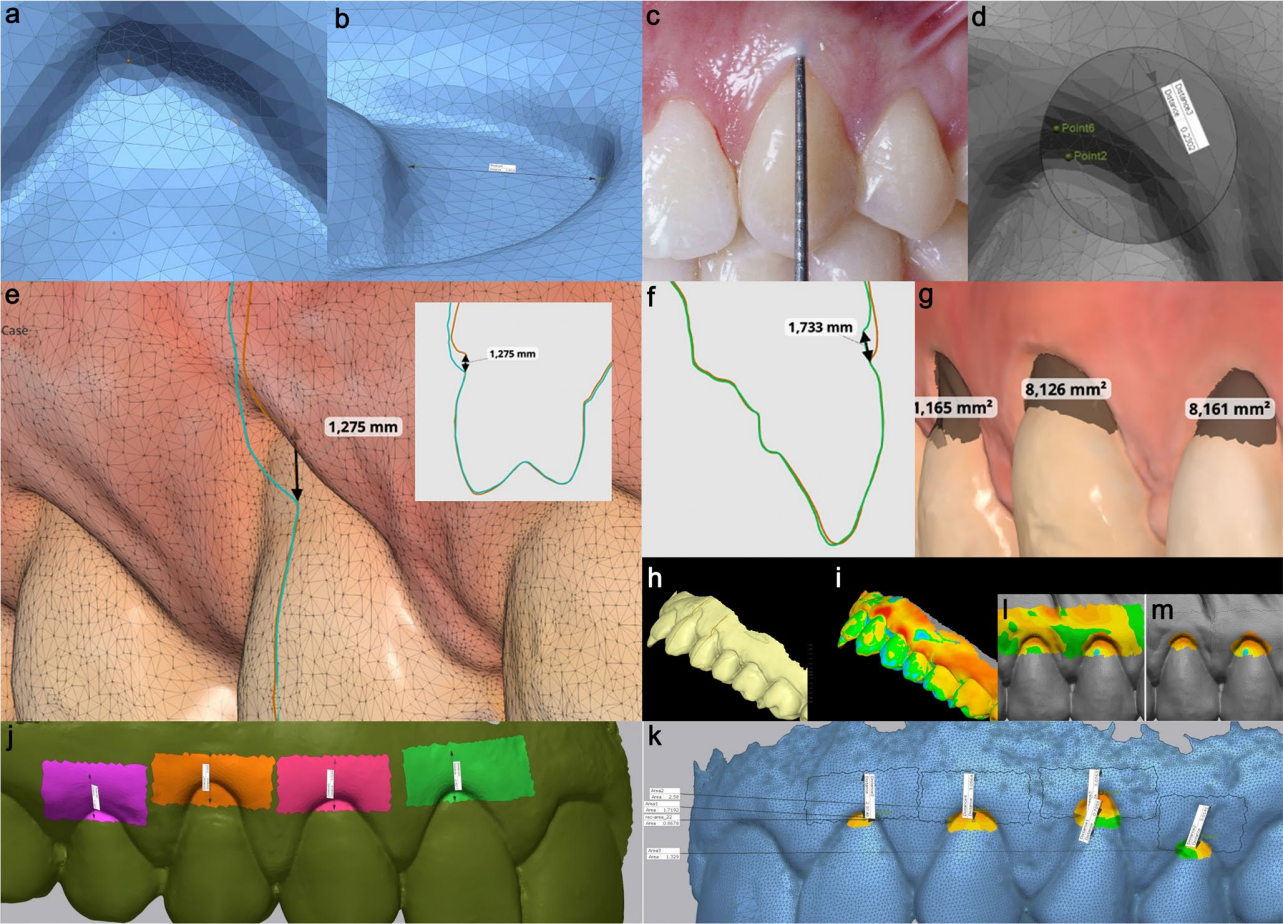
**a**,** b**. Magnification and multi-angle view in the definition of reference points and measurements; **c**,** d**. Periodontal probe scale vs. Digital measurement scale; **e**. Measurements with superimposing of models, allowing the use of the same reference point at different times; **f**,** g**. Measurement of recession depth (Re) and recession area (Re Area). Green line T0, Red line (Tf); **h**,** i**. Volume dynamics in terms of 2D (section) and 3D evaluation; **j**. Region of interest-1 (ROI-1) for a group of teeth; **k**. ROI-2 for a group of teeth; **l**,** m**. Visual evaluation of volume difference for ROI-1 and ROI-2, respectively

Assessments utilized intraoral scanning and specialized software (Medit Link and Geomagic Control X) [28], offering: (1) precision: software tools offer 10 to 100 times higher resolution than the millimetric scale of manual probes (Fig. 1.a, b); (2) accuracy: 3D optical scanning avoids soft-tissue compression and reduces inter-operator variability (Fig. 1.c, d) [29]; and (3) volume analysis: 3D superimposition allows for precise volumetric and 2D-section measurements without the radiation risks associated with CBCT scans Fig. 1.e, f,g, h,i) [17, 30].

#### STL/PLY acquisition (digital model) and region of interest (ROI)

Digital impressions (Standard Tessellation Language [STL]/Polygon File Format [PLY]) were obtained at baseline (T0) and six months (T6) using a Medit i700 scanner. Medit Link software was used to quantify linear recession depth, recession area, and CRC. Volumetric and tridimensional (3D) thickness analyses were conducted using Geomagic Control X, following the protocol of Marques et al. [17, 30].

Key 3D metrics included mean and maximum gains in GT and volumetric changes within two precisely defined ROI. ROI-1 (Fixed Tissue Area): this ROI was demarcated by the following four margins: (1) the coronal margin, defined by the 6-month gingival margin; (2) the apical margin, defined by a parallel line located 3 mm apical to the 6-month gingival margin; and (3) the lateral margins, defined by vertical lines passing through the mesiodistal papilla midpoints (Fig. 1.j, k,l, m).

ROI-2 (RC Zone): a second, semi-lunar ROI was defined to isolate the tissue formed specifically over the previously denuded root surface. Its margins were demarcated by: (1) the coronal border, corresponding to the 6-month gingival margin; (2) the apical border, corresponding to the baseline (T0) gingival margin; and (3) the lateral borders, corresponding to the mesial and distal limits of the recession defect (Fig. 1.k, m).

Within these polygonal zones, 3D superimposition quantified the mean and maximum gains in GT, focusing on the thickness of the new tissue relative to the baseline root surface (meanGT(R) and maxGT(R)).

### Preoperative intervention

Patients underwent a thorough diagnostic evaluation, followed by professional prophylaxis and instructions on appropriate brushing without causing trauma (roll technique) (NBMS). Surgical treatment of GR defects was deferred until the patient could maintain satisfactory plaque control (< 20%). After four weeks, the eligibility for the intervention was verified by a single experienced operator (NBMS).

When the cement-enamel junction (CEJ) was undetectable or showed a step [31], CEJ restoration was performed prior to the surgical procedure and digital scan (T0).

### Blood collection and membrane preparation (test group)

L-PRF preparation followed the technique described by Choukroun et al. [11]. Venous blood was collected into 4–6 glass-coated plastic tubes (9 mL) and immediately centrifuged (Intra-Lock^®^, U.S.A.) at 2700 RPM (RCF-clot = 408 g) for 12 min. The centrifugation parameters included a rotor angle of 33° and a radius of 50 mm at the clot (80 mm maximum).

Post-centrifugation, the fibrin clots were separated from the red blood cell fraction using toothed tweezers and placed on a sterile metal surface. The clots were then gently compressed by gravity using the Xpression™ (Intra-Lock, U.S.A.) box lid to produce standardized L-PRF membranes. To ensure research reproducibility, six parameters were recorded [32]: rotor dimensions (50–80 mm), 33° angulation, 2700 RPM for 12 min, RCF-clot (408 g), tube composition (9mL glass-coated plastic), and the specific centrifuge model.

### Surgical procedure

**Fig. 2:**
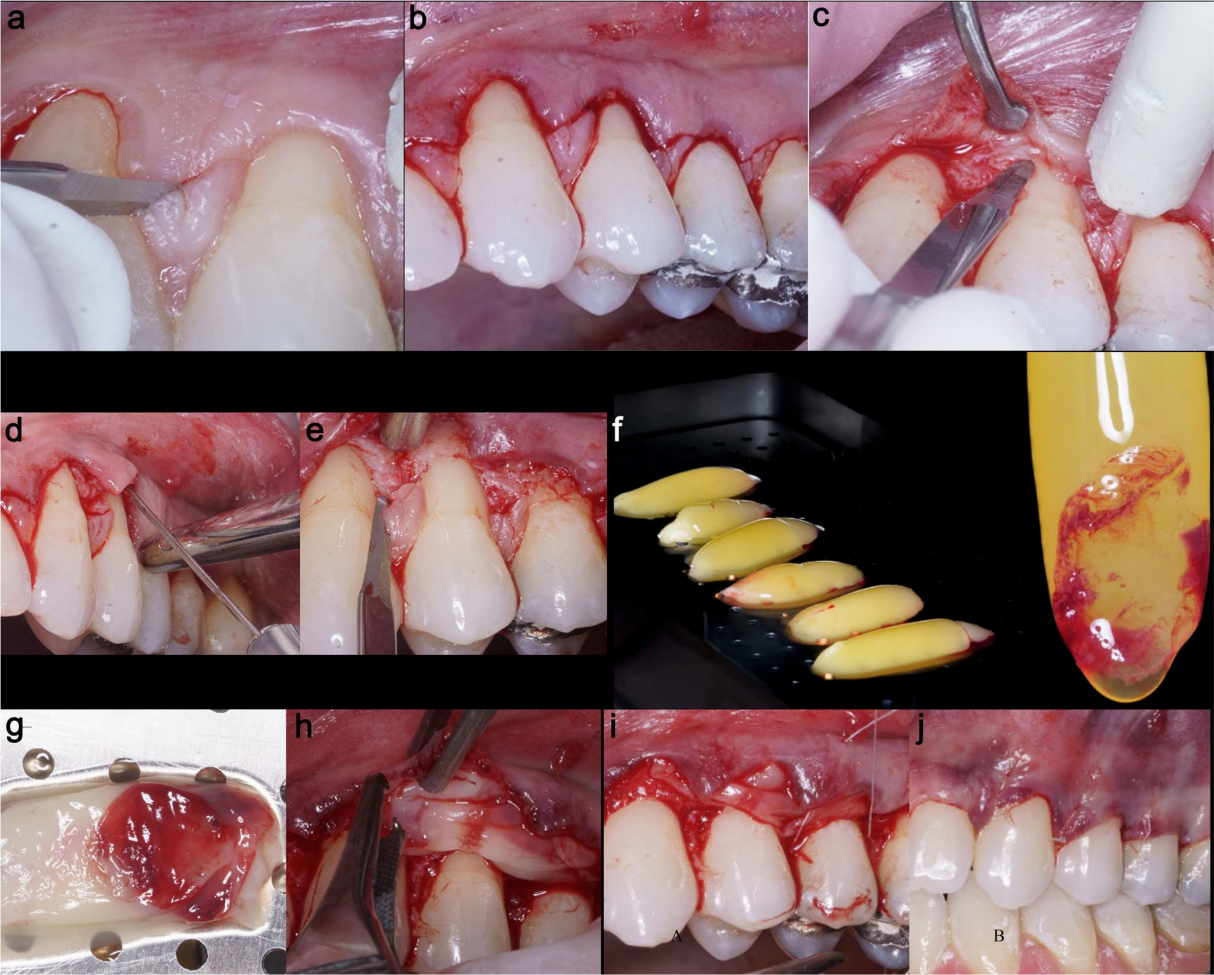
**a**. Incision creating the “surgical papilla”; **b**,** c**. Envelope flap with horizontal incisions and split flap preparation; **d**,** e**. Coronal incision for flap detachment and de-epithelialization of papillae; **f**,** g**,**h**. L-PRF-blood clot prepared, sutured together, and insertion of the L-PRF membranes (test group); **i**,** j**. Reposition of the flap and suture with a single sling suture (PTFE)

The CAF technique for multiple recession defects [33] utilized a minimally invasive envelope flap without vertical releasing incisions to optimize blood supply and esthetics [8]. Following anesthesia, horizontal oblique submarginal incisions created surgical papillae, joined by intrasulcular incisions from the central incisor to the molar. A split-full-split thickness flap was elevated: split-thickness at the interdental papillae, full-thickness at the root exposures for increased tissue bulk, and split-thickness apically beyond the mucogingival junction. Hemi-tunneling at the flap extremities and sharp periosteal dissection eliminated muscle tension, ensuring tension-free coronal displacement (Fig. 2) [34].

Anatomical papillae were de-epithelialized, and roots were instrumented with Mini-Gracey curettes. In the CAF + L-PRF group, 4–6 L-PRF membranes were sutured together and adapted to the CEJ. The flap was repositioned coronally and rotated toward the canine, securing the surgical papillae over the de-epithelialized beds. Stability was maintained with 6/0 polytetrafluoroethylene (PTFE) sling sutures [35], which were used to adapt the flap precisely to the root surfaces (Fig. 2).

### Postoperative control and follow-up

Each patient received a written document with detailed postoperative instructions. Postoperative pain and edema were controlled with ibuprofen 600 mg every 12 h for 3 days, with additional doses only if pain persisted. Patients were instructed not to brush their teeth in the treated area but to rinse their mouths with chlorhexidine solution (0.12%) twice daily for 14 days, for 1 min each time.

**Fig. 3:**
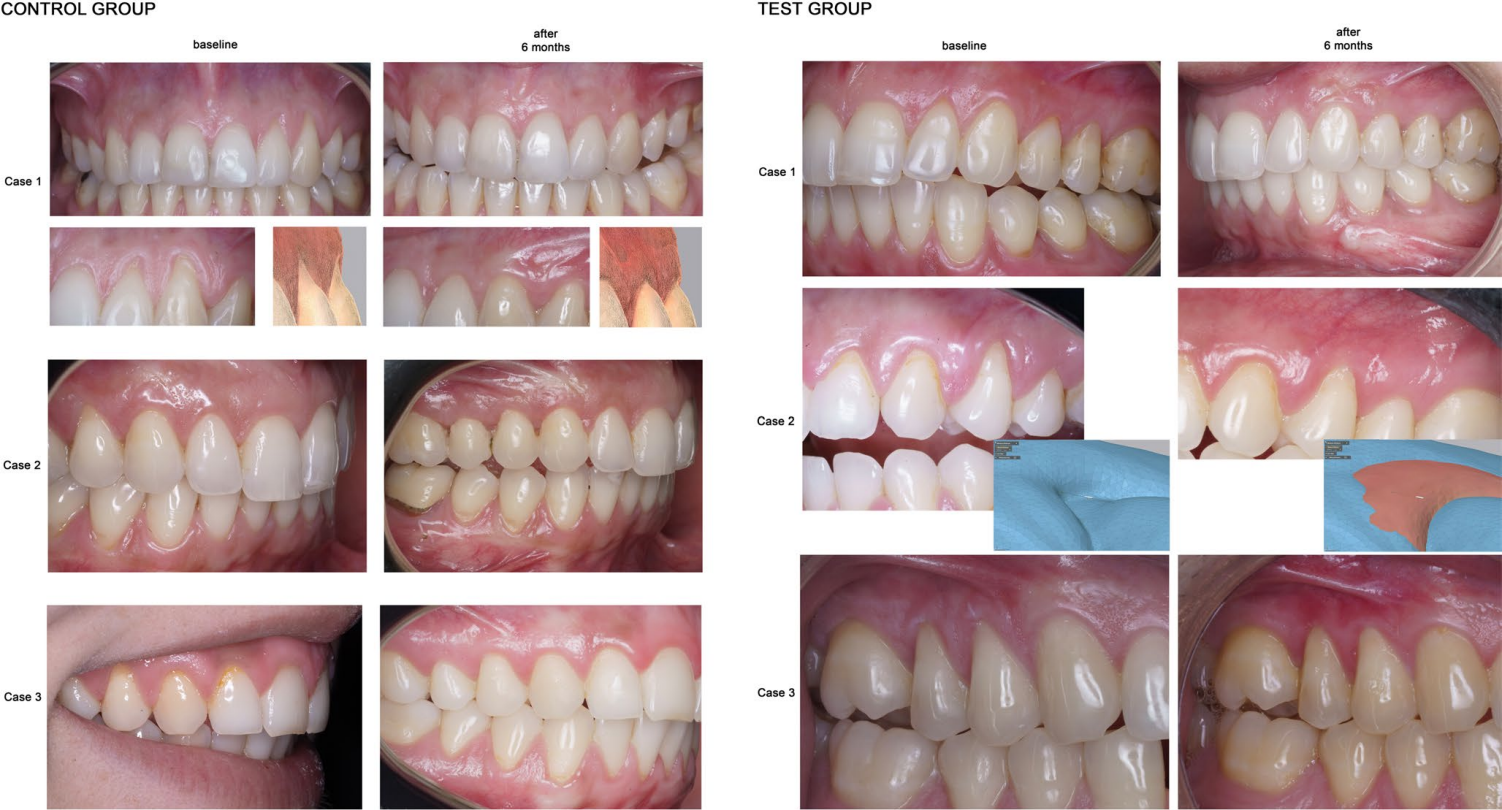
Three cases for both groups are presented. The left side shows the control group – CAF alone (one of the cases with digital evaluation); The right side presents the test group – CAF + PRF (one of the cases presenting digital evaluation)

Fourteen days after surgery, the sutures were removed. Plaque control in the surgically treated area was maintained by chlorhexidine in the first two weeks. After this period, the patients were again instructed in mechanical tooth cleaning of the treated region using a post-surgical toothbrush and a roll technique. All patients were recalled for prophylaxis with an air-polishing device at 45 days and 3 months post-intervention prior to the final 6-month evaluation. At 6 months, data were collected according to protocol (Fig. 3).

### Statistical analysis

Statistical analysis was performed using Statistical Package for the Social Sciences (SPSS) (IBM, v. 26.0) at the 5% significance level. Descriptive statistics included frequency distributions for categorical variables and measures of central tendency for continuous data.

In the inferential analysis, the primary analysis utilized a Random-Intercept Mixed-Effects Model to account for site-level clustering within patients. Normality assumptions were verified using the Shapiro-Wilk test. For patient-level data, standard inferential tests (ANOVA/Mann-Whitney) were used. No adjustments for multiple comparisons were made for the primary outcome. For patient-level data, multiple linear regression with stepwise selection (forward and backward) identified predictors for gingival thickness and volume gains.

## Results

### Sample characterization

Initially, 48 patients were screened; therefore, 26 patients did not meet the inclusion criteria (2 due to the presence of a prosthetic crown on experimental teeth, 22 because of the BoP ≥ 25%, and 4 patients had the gingival recessions with ≤ 1.5 mm of KT apical to the recession area), and 3 declined to participate. Then, nineteen patients were enrolled, with a total of 70 GRs (males had 22 [31.43%] GR and females 48 [68.57%]) (Suppl. Figure 1). They were randomly assigned to the test group (TG: 10 participants; 42 GR) and the control group (CG: 9 participants; 28 GR). The mean age was 33.32 ± 10.08 years (minimum: 19 years; maximum: 51 years) (Table 1/Suppl. Table 1).

**Table 1:** Patient-centered baseline variables comparison

Variable	Control Group (*n* = 9 patients)	Test Group (*n* = 10 patients)	*p*-value
Patient-level data			
Total Recession Sites (n)	28	42	—
Age at Surgery (years)	34.0 ± 10.2	33.6 ± 10.8	0.87
Gender (Female/Male)	7/2	7/3	1
Phenotype (Thin/Thick)	6/3	7/3	1
***Site-level baseline metrics***			
Recession Depth (mm)	1.25 ± 0.79	1.18 ± 0.66	0.68
Recession Area (mm²)	3.91 ± 2.74	3.54 ± 2.03	0.51
Keratinized Tissue Width (mm)	3.00 ± 1.01	2.74 ± 0.84	0.25

At the patient and site levels, randomization was effective in producing comparable groups. No statistically significant differences were observed between the control (CAF alone) and test (CAF + L-PRF) groups in demographic variables, gingival phenotype, recession characteristics, keratinized tissue width (KTW), or baseline sensitivity. Continuous variables, including age, baseline KTW, recession depth (ReT0), and recession area (ReA0), did not differ between groups (all *p* > 0.05), as did categorical variables such as phenotype, CEJ classification, STEP presence, and sensitivity status (Table 1/Suppl. Table 1).

### Primary outcomes: %RC and CRC

RC outcomes demonstrated high clinical success in both groups, with median %RC values of 100%. For %RC by length, the test group achieved a mean of 89.30 ± 20.33% compared to 81.60 ± 27.93% in the control group (*p* = 0.31; 95% CI: [−4.14, 19.54]) (Table 2). The Cohen’s *d* of 0.32 represents a small clinical effect size. Similarly, CRC was achieved in 73.81% of test sites versus 57.14% of control sites, representing a small-to-medium effect size (*d* = 0.35) that did not reach statistical significance (*p* = 0.19) after adjusting for patient-level clustering.

**Table 2:** Percentage of GR coverage, mean root coverage (MRC), and complete root coverage (CRC)

Outcome Metric	Group	Mean ± SD	Median	95% CI (Diff)	Cohen’s d	*p*-value (Mann-W)	*p*-value (Mixed)
GR Length (%RC)*	Control	81.60 ± 27.93	100	[−4.14, 19.54]	0.32	0.15	0.31
	Test	89.30 ± 20.33	100				
GR Area (%RC)	Control	79.46 ± 30.27	100	[−4.96, 21.42]	0.31	0.18	0.631
	Test	87.69 ± 23.48	100				
MRC (mm)	Control	0.949 ± 0.680	0.685	[−0.25, 0.36]	0.09	0.26	0.81
	Test	1.005 ± 0.587	0.835				
CRC (%)	Control	57.14% (*n* = 16)	—	[1.02, 32.32]**	0.35†	0.199‡	0.193
	Test	73.81% (*n* = 31)	—				

*Primary Outcome. **95% CI for the difference in proportions. †Calculated as d equivalent. ‡Chi-square test p-value

### Secondary outcomes

#### MRC and %RC by Area

MRC and %RC-area followed a similar pattern of numerical improvement in the test group without statistically significant intergroup differences (*p* > 0.25) (Table 2). Specifically, %RC-area was 87.69 ± 23.48% in the test group versus 79.46 ± 30.27% in the control group (*p* = 0.63; *d* = 0.31). Analysis of MRC showed near-identical results between groups (1.01 ± 0.59 mm for Test vs. 0.95 ± 0.68 mm for Control; *p* = 0.81; *d* = 0.09).

#### KTW

Both groups demonstrated a gain in KTW at the 6-month follow-up. The mean increase was 0.29 mm for the test group and 0.06 mm for the control group. This difference was not statistically significant (*p* = 0.31; *d* = 0.21) after accounting for dependency between multiple sites (Table 3).

**Table 3:** Statistical analysis and comparative data for ΔGT and DKTW, and VAS results

Part A: Volumetric and Gingival Thickness (GT) Changes at 6 Months.							
Variable	Group	Min – Max	Mean ± SD	Median	95% CI (Diff)	Cohen’s d	Mann-Whitney (*p*)
Mean ΔGT (ROI 1)	Control	−0.14–0.28	0.11 ± 0.10	0.12	[0.00, 0.10]	0.48	−0.78 (0.075)
	Test	−0.05–0.37	0.16 ± 0.11	0.15			
Max ΔGT (ROI 1)	Control	0.25–1.11	0.55 ± 0.20	0.55	[−0.08, 0.14]	0.13	−0.18 (0.857)
	Test	0.19–1.46	0.58 ± 0.27	0.55			
Mean ΔGT (ROI 2)	Control	0.01–0.61	0.30 ± 0.16	0.3	[−0.05, 0.11]	0.18	−0.43 (0.670)
	Test	0.02–0.77	0.33 ± 0.17	0.28			
Max ΔGT (ROI 2)	Control	0.11–1.11	0.51 ± 0.23	0.54	[−0.15, 0.47]	0.25	−0.11 (0.914)
	Test	0.15–6.40	0.67 ± 0.93	0.49			
Volume in ROI 2	Control	0.00–3.05	0.86 ± 0.84	0.71	[−0.25, 0.79]	0.25	−0.82 (0.415)
	Test	0.05–6.52	1.13 ± 1.25	0.64			
Part B: Linear Mixed-Effects Model for Gingival Thickness and Volume.							
Variable	**Intercept (Coef.)**	**Group Coef.**	**p-value (Group)**	**Group Var.**	**Residual Var.**	**Log-Likelihood**	
Mean ΔGT (ROI 1)	0.111 (*p* < 0.001)	0.05	0.106	0.002	0.0089	55.546	
Max ΔGT (ROI 1)	0.547 (*p* < 0.001)	0.04	0.536	0.003	0.0561	−3.785	
Volume (ROI 2)	0.841 (*p* < 0.001)	0.314	0.346	0.228	1.0006	−105.089	
Part C: Keratinized Tissue Width Gain (ΔKTW).							
Metric	**KTW T0 (mm)**	**KTW 6 m (mm)**	**Mean Change**	**95% CI (Diff)**	**Cohen’s d**	**p-value (Mixed)**	
Mean ± SD	2.85 ± 0.91	3.04 ± 0.89	+ 0.19 mm	[−0.21, 0.59]	0.21	0.307	
Part D: Patient-Reported Outcome Measures (PROMs) at 5 Days.							
Variable	**Group**	**Mean ± SD**	**Median**	**95% CI (Diff)**	**Cohen’s d**	**Mann-Whitney (p)**	
VAS 5 days	Control	1.22 ± 1.09	1	[−0.63, 0.99]	0.14	0.01 (0.999)	
	Test	1.40 ± 1.51	1				
Pills (Analgesics)	Control	3.11 ± 2.57	3	[−3.18, 0.36]	0.68	−1.22 (0.243)	
	Test	1.70 ± 1.25	1				

#### Gingival thickness and volumetric outcomes

Volumetric analysis revealed a numerical trend toward greater tissue augmentation in the CAF + L-PRF group. For the mean ΔGT (ROI 1), the test group achieved a gain of 0.16 ± 0.11 mm compared to 0.11 ± 0.10 mm in the control group (*p* = 0.08). The Cohen’s *d* of 0.48 indicates a moderate clinical effect size for tissue thickening in this region (Table 3). Maximum thickness gain (Max ΔGT) in ROI 2 was 0.67 ± 0.93 mm for the test group and 0.51 ± 0.23 mm for the control group (*p* = 0.35; 95% CI: [−0.15, 0.47]; *d* = 0.25). Mean Volume Gain in ROI 2 was 1.13 ± 1.25 mm^3^ (Test) versus 0.86 ± 0.84 mm^3^ (Control), resulting in a small effect size (*d* = 0.25) that was not statistically significant (*p* = 0.35).

#### Healing, pain, postoperative morbidity, and Patient-reported outcome measures (PROMs)

**Fig. 4:**
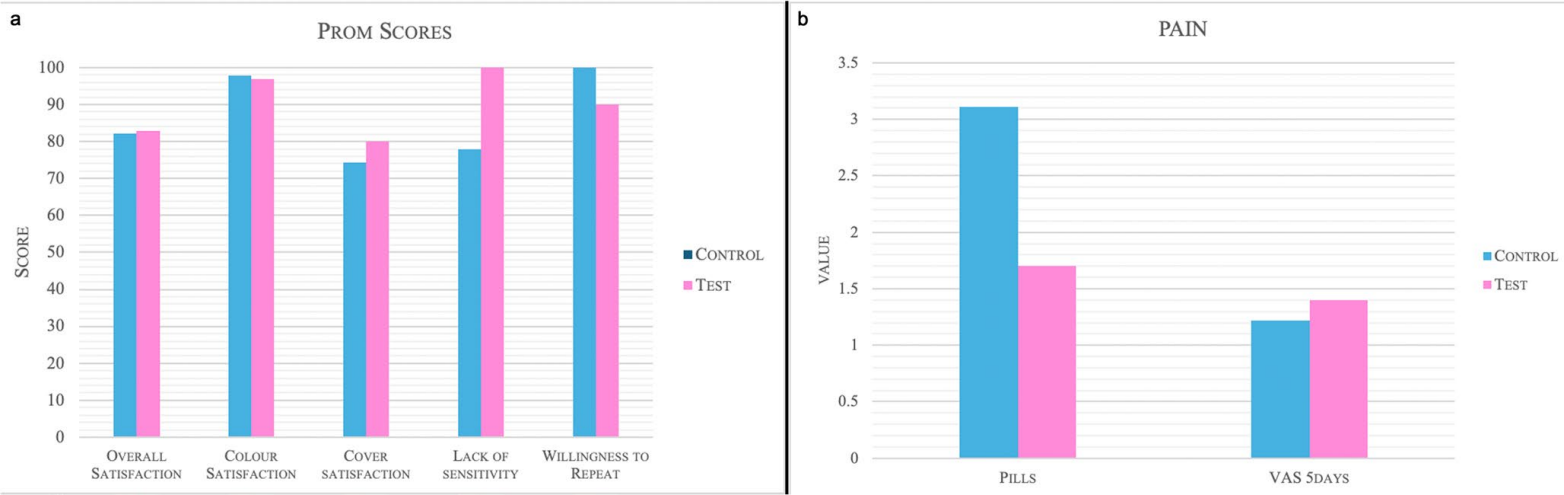
**a**. PROMs scores; **b**. Pill consumption and VAS score at 5 days

Healing outcomes (IPR scale) showed comparable inflammatory, proliferative, and remodeling responses across groups (*p* > 0.05). Postoperative pain (VAS) and analgesic consumption were low in both groups (Fig. 4; Table 3), with no statistically significant differences. PROMs revealed high satisfaction for color match, perceived recession coverage, and overall treatment satisfaction (*p* > 0.05). Most patients expressed a willingness to undergo the procedure again, reinforcing the high patient acceptance of both surgical protocols (Fig. 4).

## Discussion

The primary clinical objective of this study was to evaluate %RC and CRC. Our results indicate that adjunctive L-PRF did not yield statistically significant improvements in these primary outcomes compared with CAF alone. Furthermore, secondary outcomes (including MRC, GT, Vol gain, and PROMs) mirrored this parity, showing no significant intergroup differences. These findings are consistent with systematic reviews suggesting that in small-to-moderate RT1 recessions, the incremental benefits of platelet concentrates may not reach statistical significance [13, 36, 37]. While L-PRF provides a bioactive scaffold rich in growth factors intended to enhance healing [38], our data suggest that in this specific clinical scenario, these biological properties did not translate into superior primary or secondary clinical outcomes.

The effectiveness of the randomization process was confirmed by the baseline comparability of demographic and clinical variables (*p* > 0.05), ensuring that outcomes were attributable to the interventions rather than to initial imbalances. In the present study, the test group (CAF + L-PRF) showed a numerically higher proportion of CRC (73.8% vs. 57.1%) and a higher MRC, but these differences did not reach statistical significance (*p* > 0.20). These results align with prior literature suggesting that adjunctive biomaterials often yield only incremental gains in smaller cohorts [37, 39]. While meta-analyses have suggested a potential 10–15% improvement with PRF-based approaches [40], the clinical relevance of this magnitude remains uncertain in RT1 defects, where CAF alone already performs at a high level [41].

Regarding soft-tissue changes, both groups showed increased KTW at six months. The slightly higher gain in the test group was not statistically significant (*p* > 0.17), corroborating evidence that L-PRF does not significantly enhance KTW when combined with CAF [37]. Similarly, although baseline GT and volume are critical predictors of long-term stability [41–43], our volumetric analysis revealed no significant intergroup differences across all ROIs (all *p* > 0.10). While some studies report GT gains with L-PRF, others report mixed results influenced by membrane resorption and technique-related factors [44].

A distinct feature of this study was the use of a digital-assisted workflow. Traditional transgingival probing is limited by low resolution and examiner variability [45, 46]. Using 3D imaging and digital superimposition, we achieved a resolution on the order of hundredths of a millimeter without the risk of tissue trauma or compression [47]. This precise methodology confirmed that, even with four to six membranes used to maximize biological density [35, 48], the differences in tissue volume between the two groups remained non-significant.

Regarding healing responses, postoperative morbidity was comparable across groups. Postoperative pain was low, and although the test group reported lower analgesic consumption, the difference was not significant. These findings confirm that adding L-PRF is well tolerated but does not markedly alter the patient’s postoperative experience compared with CAF alone [44]. In summary, while L-PRF is biologically plausible due to its sustained release of growth factors [35], its clinical advantage in treating small RT1 recessions remains modest and statistically unproven in this cohort [37].

### Clinical relevance and minimal clinically important difference (MCID)

A critical consideration in interpreting these results is the clinical context of RT1 defects. RT1 is associated with a high probability of CTC using CAF alone, creating a “ceiling effect” that strictly limits the detectable incremental benefit of any adjunctive therapy. In this study, despite using a digital workflow that provided submillimetric resolution, intergroup differences in soft-tissue volume remained statistically nonsignificant. This raises the important clinical issue of potential overtreatment. Because CAF remains highly predictable and effective for RT1 defects, the routine addition of L-PRF increases surgical complexity, chair time, and patient burden without delivering proportional clinical benefits.

This raises the important clinical question of potential overtreatment. Since CAF remains highly predictable for RT1 defects, the routine addition of L-PRF could be viewed as an unnecessary increase in surgical complexity and chair time. However, the MCID for volumetric soft-tissue augmentation has not yet been standardized, and the numerical increases observed in the L-PRF group may have long-term structural value. Previous longitudinal data suggest that modest phenotype modification, shifting from a thin to a thicker gingival profile, is a primary predictor of 10-year stability and resistance to recession relapse. Therefore, while the immediate clinical gain of L-PRF appears limited in small RT1 defects where CAF is already highly effective, its contribution to the structural “bulk” of the gingival margin may offer protective benefits against future relapse. The use of L-PRF should thus be balanced against the clinical goals: if the objective is simply immediate RC, CAF alone is sufficient; if the goal is long-term phenotype reinforcement in a thin-biotype patient, the biological adjunct may be justified.

### Limitations of the study

Study limitations include a relatively small patient cohort, limited recession depth, and a short 6-month follow-up period. These factors likely reduced the power to detect minor differences in volumetric or clinical outcomes. Furthermore, the use of stepwise multiple linear regression on a limited sample (*n* = 19) is a methodological limitation; these findings should be interpreted as exploratory rather than definitive. Future multicenter trials with longer observation periods are warranted to determine whether L-PRF confers advantages in long-term stability or relapse resistance.

## Conclusion

Within the limitations of this study, both CAF and CAF + L-PRF achieved comparable primary and secondary clinical and volumetric outcomes at six months for the treatment of RT1 GRs. No statistically significant differences were found in the primary endpoints of RC, nor in secondary parameters such as KTW, GT, or patient satisfaction. Although the digital workflow provided high-resolution data, the adjunctive value of L-PRF in small defects is not statistically supported. Given the high success rate of CAF alone for RT1 defects, the routine addition of L-PRF in this specific clinical scenario may not be relevant.

## Supplementary Information

Below is the link to the electronic supplementary material.

Supplementary Material 1 (DOCX 15.3 KB)

Supplementary Material 2 (JPG 286 KB)

## Data Availability

No datasets were generated or analysed during the current study.
