# Supplementary material for: Treatment of gingival recession type 1 using coronally advanced flap with leucocytes-platelet rich fibrin: a randomized controlled trial
Source: Clin Oral Investig. 2026 May 7;30(6):220. doi: 10.1007/s00784-026-06899-4 (PMC13152877; doi:10.1007/s00784-026-06899-4)
Supplement: Supplementary file 1 — Supplementary Material 1 (DOCX 15.3 KB) [file 784_2026_6899_MOESM1_ESM.docx]

**Suppl. Table 1**. Patient- and site-level comparisons between groups.

| **Variable** | **Category** | **Control Group** | **Test Group** | **Statistical Marker** | **p-value** |
| --- | --- | --- | --- | --- | --- |
| ***Patient-Level Comparisons*** |  | (n=9 patients) | (n=10 patients) |  |  |
| Sensitivity | Sensitivity (+) | 3 (33.3%) | 4 (40.0%) | Odds Ratio: 0.75 | 1 |
| CEJ (Pini-Prato) | Class B | 4 (44.4%) | 7 (70.0%) | Odds Ratio: 0.34 | 0.37 |
| STEP (Pini-Prato) | STEP (+) | 4 (44.4%) | 7 (70.0%) | Odds Ratio: 0.34 | 0.37 |
| ***Site-Level Comparisons*** |  | (n=28 sites) | (n=42 sites) |  |  |
| Sensitivity | Presence (+) | 7 (25.0%) | 13 (31.0%) | Chi-Square: 0.29 | 0.79* |
| Phenotype | Thin | 21 (75.0%) | 30 (71.4%) | Chi-Square: 0.11 | 0.79* |
| CEJ (Pini-Prato) | Class A | 22 (78.6%) | 32 (76.2%) | Chi-Square: 0.05 | 1.00* |
| STEP (Pini-Prato) | STEP (-) | 21 (75.0%) | 28 (66.7%) | Chi-Square: 0.56 | 0.60* |

*p-value calculated using Fisher's Exact Test.
