# Supplementary figures and images for: Treatment of gingival recession type 1 using coronally advanced flap with leucocytes-platelet rich fibrin: a randomized controlled trial
Source: Clin Oral Investig. 2026 May 7;30(6):220. doi: 10.1007/s00784-026-06899-4 (PMC13152877; doi:10.1007/s00784-026-06899-4)

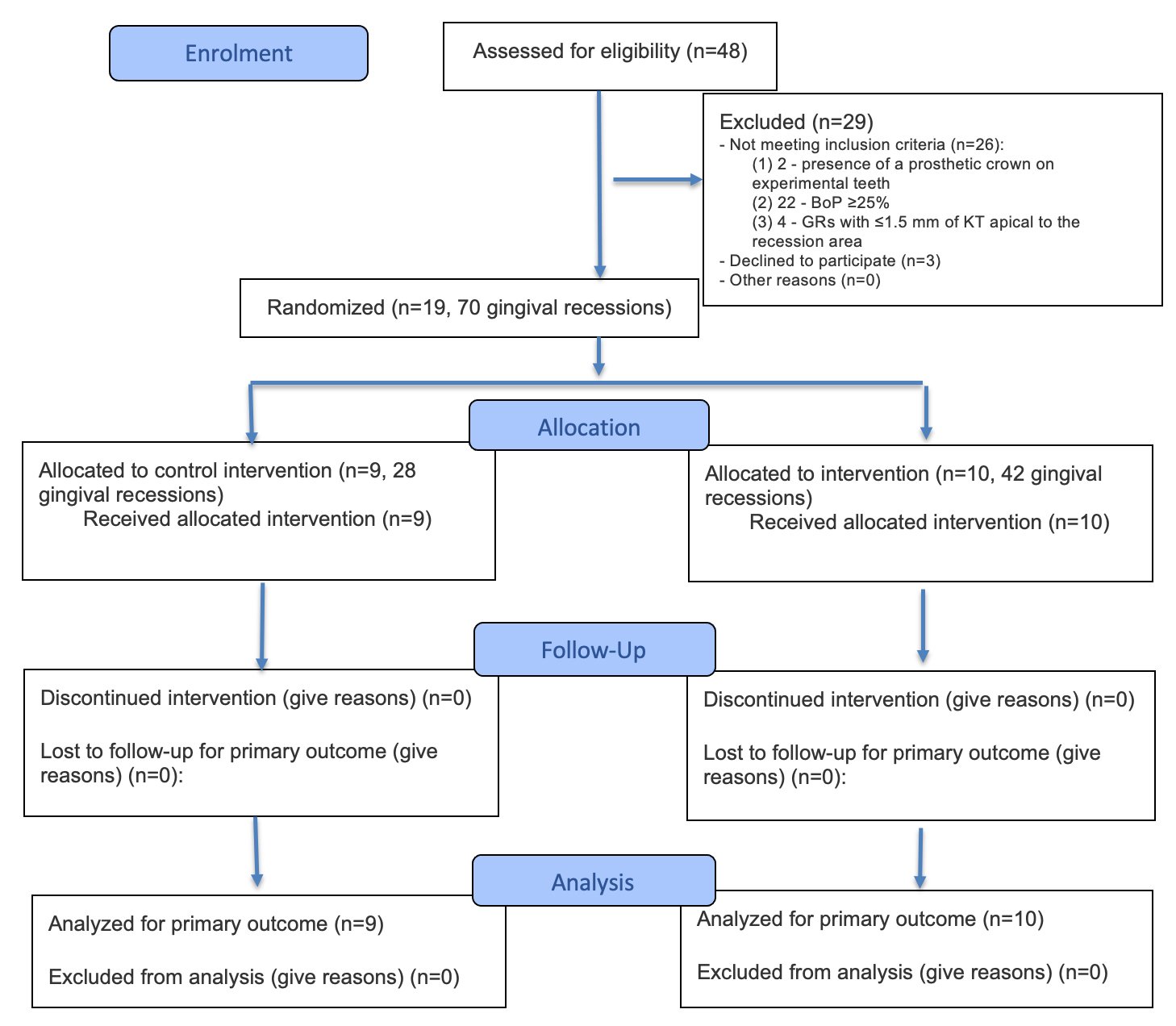

Supplement: Supplementary file 2 — Supplementary Material 2 (JPG 286 KB) [file 784_2026_6899_MOESM2_ESM.jpg]
